# T Cell‐Independent Role of PD‐L1 in Kidney Repair: Mitigation of Tubular DNA Damage via PD‐L1/BRCA1 Interaction Following AKI

**DOI:** 10.1002/advs.75428

**Published:** 2026-04-27

**Authors:** Wei Jiang, Tao‐Tao Tang, Wei‐Jie Ni, Jin‐Xuan Wei, Liang‐Yun‐Zi Jiang, Qing Yin, Yi‐Lin Zhang, Zuo‐Lin Li, Yi Wen, Xin‐Lu Wang, Jun‐Yuan Shen, Xiao‐Jun Ouyang, Ming‐Zhu Zheng, Jian Xu, Xiaofei An, Lin‐Li Lv, Bi‐Cheng Liu, Bin Wang

**Affiliations:** ^1^ Institute of Nephrology Zhongda Hospital School of Medicine Southeast University Nanjing Jiangsu China; ^2^ Department of Geriatric Geriatric Hospital of Nanjing Medical University Nanjing China; ^3^ Department of Nephrology Ju Rong People's Hospital Zhenjiang China; ^4^ Department of Pathogenic Biology and Immunology Jiangsu Provincial Key Laboratory of Critical Care Medicine School of Medicine Southeast University Nanjing Jiangsu China; ^5^ Department of Intensive Care Unit Geriatric Hospital of Nanjing Medical University Nanjing China; ^6^ Department of Endocrinology The Affiliated Hospital of Nanjing University of Chinese Medicine Nanjing China

**Keywords:** AKI, BRCA1, DNA damage, extracellular vesicles, PD‐L1

## Abstract

Acute kidney injury (AKI) occurs in the patients undergoing anti‐programmed cell death protein 1‐ligand 1 (PD‐L1) therapy, indicating that PD‐L1 may play a critical role in maintaining renal homeostasis. However, the precise role and mechanism of PD‐L1 in AKI remains largely elusive. In this study, we found that PD‐L1 was primarily expressed in proximal tubules and significantly upregulated in both murine models of AKI and renal biopsy samples from AKI patients. Genetic specific deletion of PD‐L1 in mouse tubular epithelial cells (TECs) exacerbated renal injury in ischemia‐reperfusion injury‐induced AKI. Mechanistically, PD‐L1 was found to interact with BRCA1 and increase BRCA1 expression to safeguard TECs against DNA damage, thereby promoting cellular proliferation and suppressing apoptosis. To translate these findings into a potential therapeutic strategy, we developed a CGA‐functionalized extracellular vesicle delivery system for targeted delivery of PD‐L1 to injured TECs. This system efficiently restored PD‐L1 expression and alleviated DNA damage of TECs in both TEC‐specific PD‐L1 knockdown and T‐cell knockout AKI mouse models. Collectively, these findings uncover a novel function of PD‐L1 in promoting adaptive TEC repair through BRCA1 interaction, independent of its canonical immunomodulatory function of T cells, and suggest that PD‐L1 supplementation may represent a promising therapeutic strategy for AKI.

## Graphical abstract

PD‐L1 is primarily expressed in renal tubules and upregulated in both murine models of AKI and renal biopsy samples from patients with AKI. PD‐L1 can promote adaptive TECs repair through interacting with BRCA1, independent of its canonical immunomodulatory function of T cells, and PD‐L1 supplementation may represent a promising therapeutic strategy for AKI.

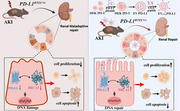

## 1 Introduction

Acute kidney injury (AKI), represented by a sharp decline in renal function, markedly increases the risks of chronic kidney disease (CKD) and end‐stage renal disease (ESRD) [1, 2]. Understanding the cellular and molecular drivers of AKI is essential for developing effective therapeutics. Tubular injury has been widely accepted as a significant contributor to AKI [3]. However, the underlying mechanisms involved in tubular injury and repair are still largely unknown.

Programmed cell death protein 1‐ligand 1 (PD‐L1, also known as CD274) is a type I transmembrane protein composed of 290 amino acids, and has been known as a crucial immune checkpoint inhibitor [4, 5]. While the clinical benefits of anti‐PD‐L1 antibodies are well‐documented, their use has been associated with renal‐related adverse events, particularly an increased risk of AKI [6, 7]. In a murine model of ischemia‐reperfusion injury (IRI)‐induced AKI, administration of anti‐PD‐L1 antibodies exacerbates renal dysfunction and tubular necrosis, suggesting a protective role of PD‐L1 in maintaining renal homeostasis [8]. However, its specific role in the kidney injury and repair processes during AKI remains poorly understood.

PD‐L1 plays a crucial role in maintaining physiological homeostasis through both T cell–dependent and T cell–independent mechanisms [9, 10]. Additionally, studies on PD‐L1 have focused on its interaction with programmed death receptor 1 (PD‐1) on T cells, where it suppresses immune responses in various contexts, including tumors, autoimmune diseases, and transplant rejection reactions [11, 12, 13]. Interestingly, emerging evidence indicates that PD‐L1 can also directly regulate cellular phenotypes–such as proliferation, metabolism, and stemness–independent of T cells, by regulating downstream gene expression in tumor cells [14, 15, 16, 17]. Our recent single‐cell RNA sequencing analysis revealed that T lymphocyte infiltration predominantly occurs during the chronic stage, rather than the early reparative stage, following AKI [18]. This finding raises the possibility that PD‐L1 may exert protective effects during the early stage of AKI through T cell‐independent pathway. Nonetheless, the underlying mechanisms still need to be further explored.

In the present study, we employed multi‐omics approaches and diverse animal models, including renal tubular‐specific PD‐L1 knockdown mice and T‐cell receptor alpha constant region knockout mice, to elucidate the role of PD‐L1 in the pathophysiology of AKI. Our findings demonstrated that PD‐L1 protects tubular epithelial cells (TECs) from DNA damage through a direct interaction with BRCA1, independent of the T cell pathway. Furthermore, we developed a targeted therapeutic platform utilizing extracellular vesicles to efficiently deliver PD‐L1 to the injured kidney, thereby alleviating DNA damage in TECs. Overall, our study underscores a novel role for PD‐L1 in safeguarding TECs and provides an effective PD‐L1 supplementation system for the treatment of AKI.

## 2 Results

### 2.1 PD‐L1 Expression is Upregulated in Mice and Patients with AKI

**FIGURE 1:**
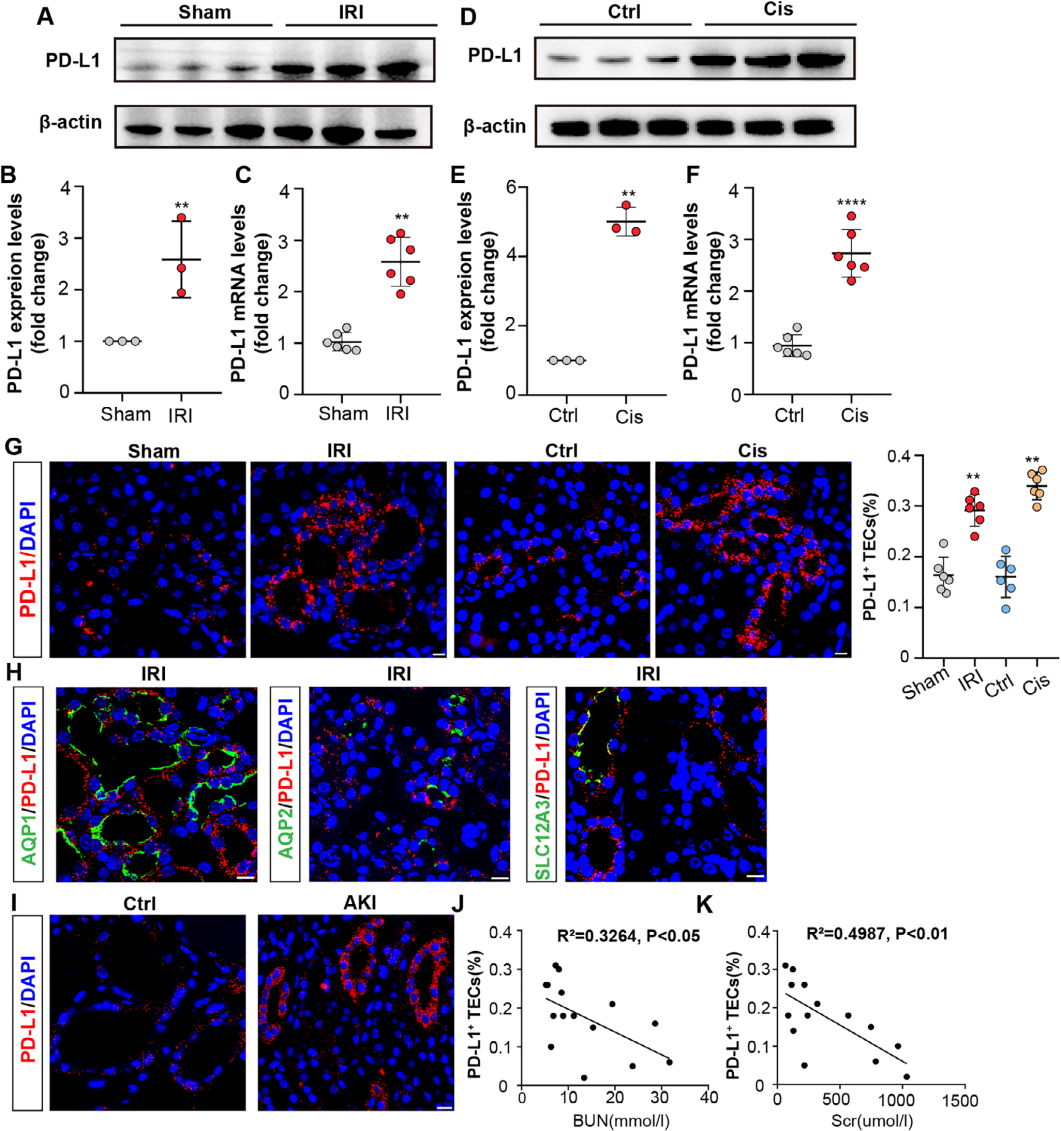
PD‐L1 expression is upregulated in mice and patients with AKI. (A) Western blot analysis of PD‐L1 protein levels in the kidneys of sham mice, murine model of renal IRI administration. (B, C) Quantifying the protein and mRNA levels of PD‐L1 in the kidneys of sham mice, murine models of renal IRI administration. (D) Western blot analysis of PD‐L1 protein levels in the kidneys of murine model of saline or cisplatin (Cis) administration. (E, F) Quantifying the protein and mRNA levels of PD‐L1 in the kidneys of murine model of saline or Cis administration. (G) Representative immunofluorescence images of the positive area of PD‐L1 staining in sham mice, murine models of renal IRI and Cis administration. (H) Representative immunofluorescence images of the location of PD‐L1 and with AQP1, SLC12A3 or AQP2 in renal IRI murine model. (I) Representative immunofluorescence images of the positive area of PD‐L1 staining in kidney biopsies from AKI patients and the normal people. (J, K) The relationship between PD‐L1‐positive area with BUN or Scr levels in kidney biopsies from AKI patients. Scale bars, 20 µm. Data are presented as means ± SD. **P < 0.01, **** *P* < 0.0001 compared to sham or control group.

Murine models of renal IRI and cisplatin (Cis) administration were established to explore the expression pattern of PD‐L1 following AKI. Western blot and qPCR results revealed a robust increase of PD‐L1 expression in both IRI‐ and Cis‐treated kidneys (Figure 1A–F). Immunofluorescence staining confirmed this upregulation, with PD‐L1 signals predominantly localized to renal tubules (Figure 1G). Co‑staining with segment‑specific markers further demonstrated strong co‐localization of PD‐L1 with AQP1 (a proximal tubule marker), but not with SLC12A3 (a distal tubule marker) or AQP2 (a collecting duct marker), indicating that PD‐L1 is primarily upregulated in the cytoplasm of proximal tubular epithelial cells (PTECs) (Figure 1H). In parallel, kidney biopsies from AKI patients also exhibited elevated PD‐L1 expression (Figure 1I), and the percentage of PD‐L1‐positive area was negatively correlated with blood urea nitrogen (BUN) and serum creatinine (Scr) levels (Figure 1J,K), suggesting a potential protective role of PD‐L1 in AKI.

To assess the relationship between injury severity and PD‐L1 expression, a series of IRI models with increasing ischemia durations (20, 28, and 35 mins) were constructed. We found that prolonged ischemia led to more severe renal injury, accompanied by a gradual decline in PD‐L1 expression (Figure S1). These findings demonstrated that PD‐L1 is expressed in PTECs and upregulated in response to AKI, but its expression diminishes with increasing injury severity.

### 2.2 PD‐L1 Promotes the Repair of Injured TECs After Hypoxia/Reoxygenation In Vitro

**FIGURE 2:**
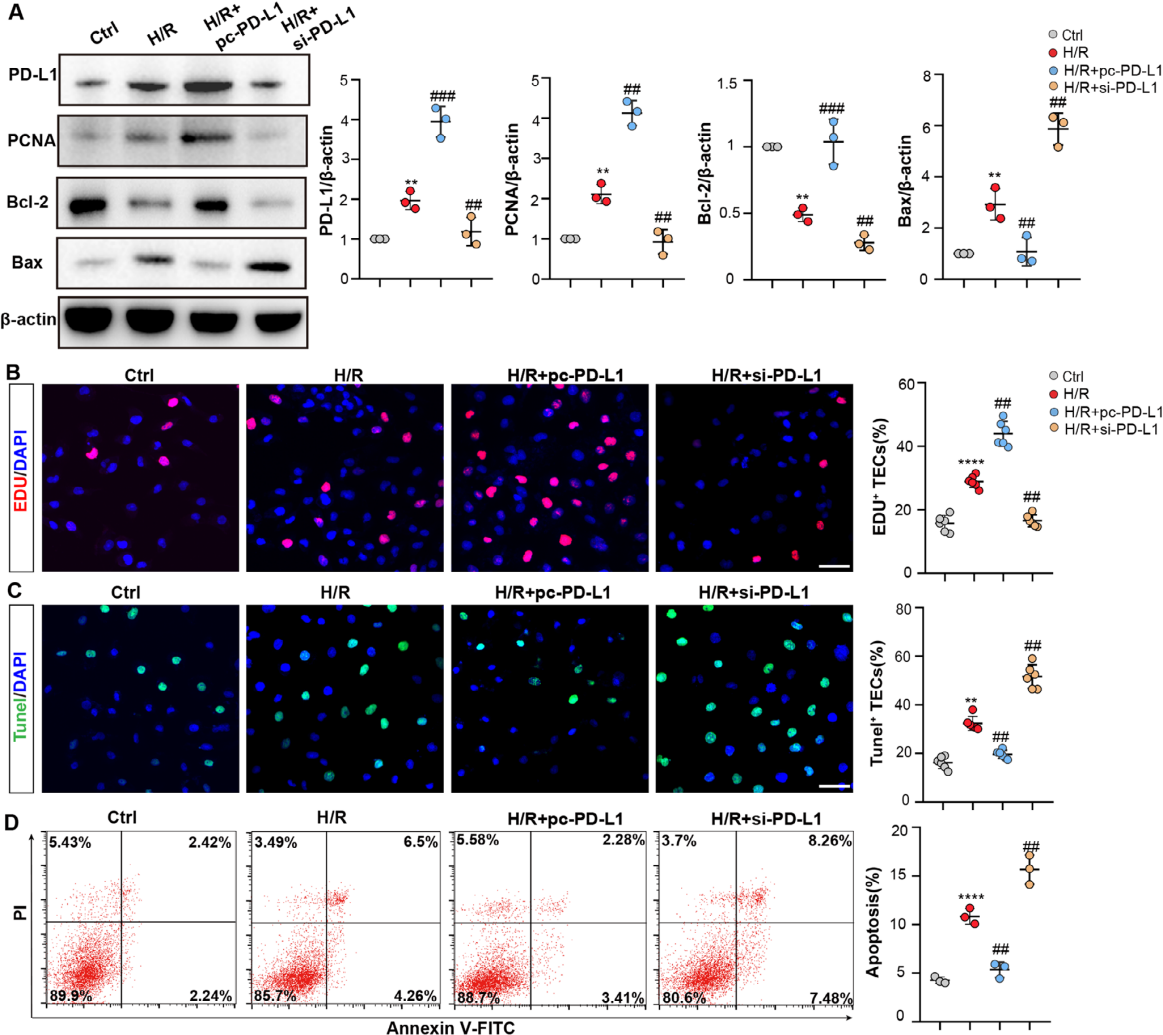
PD‐L1 promotes the repair of injured TECs after hypoxia/reoxygenation in vitro. (A) Western blot analysis of PD‐L1, PCNA, Bcl‐2 and Bax protein levels in the transfecting mTECs with either si‐PD‐L1 or pc‐PD‐L1 after H/R treatment. (B, C) EDU, Tunel staining analysis of cell proliferation and apoptosis in the transfecting mTECs with either si‐PD‐L1 or pc‐PD‐L1 after H/R treatment. (D) Flow cytometer analysis of cell apoptosis in the transfecting mTECs with either si‐PD‐L1 or pc‐PD‐L1 after H/R treatment. Scale bars, 20 µm. Data are presented as means ± SD. **P < 0.01, **** *P* < 0.0001 compared to control group; ^##^ *P* < 0.01, ^###^ *P* < 0.001 compared to mTECs after H/R treatment.

To reveal the functional role of PD‐L1 in vitro, primary murine TECs (mTECs) were isolated and cultured as previously reported [19]. PD‐L1 expression was modulated by transfecting mTECs with either PD‐L1 siRNA (si‐PD‐L1) or PD‐L1 overexpressing plasmids (pc‐PD‐L1). The transfected mTECs were then subjected to hypoxia/reoxygenation (H/R) to mimic ischemic AKI. H/R treatment elevated the expression of PCNA and Bax while reducing Bcl‐2 levels, indicating enhanced proliferative and apoptotic responses (Figure 2A). Notably, PD‐L1 knockdown significantly inhibited proliferation and promoted apoptosis in mTECs, whereas PD‐L1 overexpression reversed these effects (Figure 2A). The role of PD‐L1 in cellular proliferation and apoptosis was further supported by EDU, Tunel staining, and flow cytometer analysis (Figure 2B‐D).

Subsequently, we further validated these findings in human HK‐2 cells, which were transfected with pc‐PD‐L1 or si‐PD‐L1 and exposed to H/R. Western blot confirmed successful modulation of PD‐L1 expression (Figure S2A,B). Consistent with the mTECs results, PD‐L1 knockdown suppressed proliferation and induced apoptosis in HK‐2 cells, while PD‐L1 overexpression upregulated proliferation and reduced apoptosis (Figure S2C–F). These findings demonstrated that PD‐L1 protects injured TECs from H/R damage by stimulating cell proliferation and suppressing apoptosis in vitro.

### 2.3 Selective Knockdown of PD‐L1 in Renal Tubules Exacerbates IRI‐Induced AKI

**FIGURE 3:**
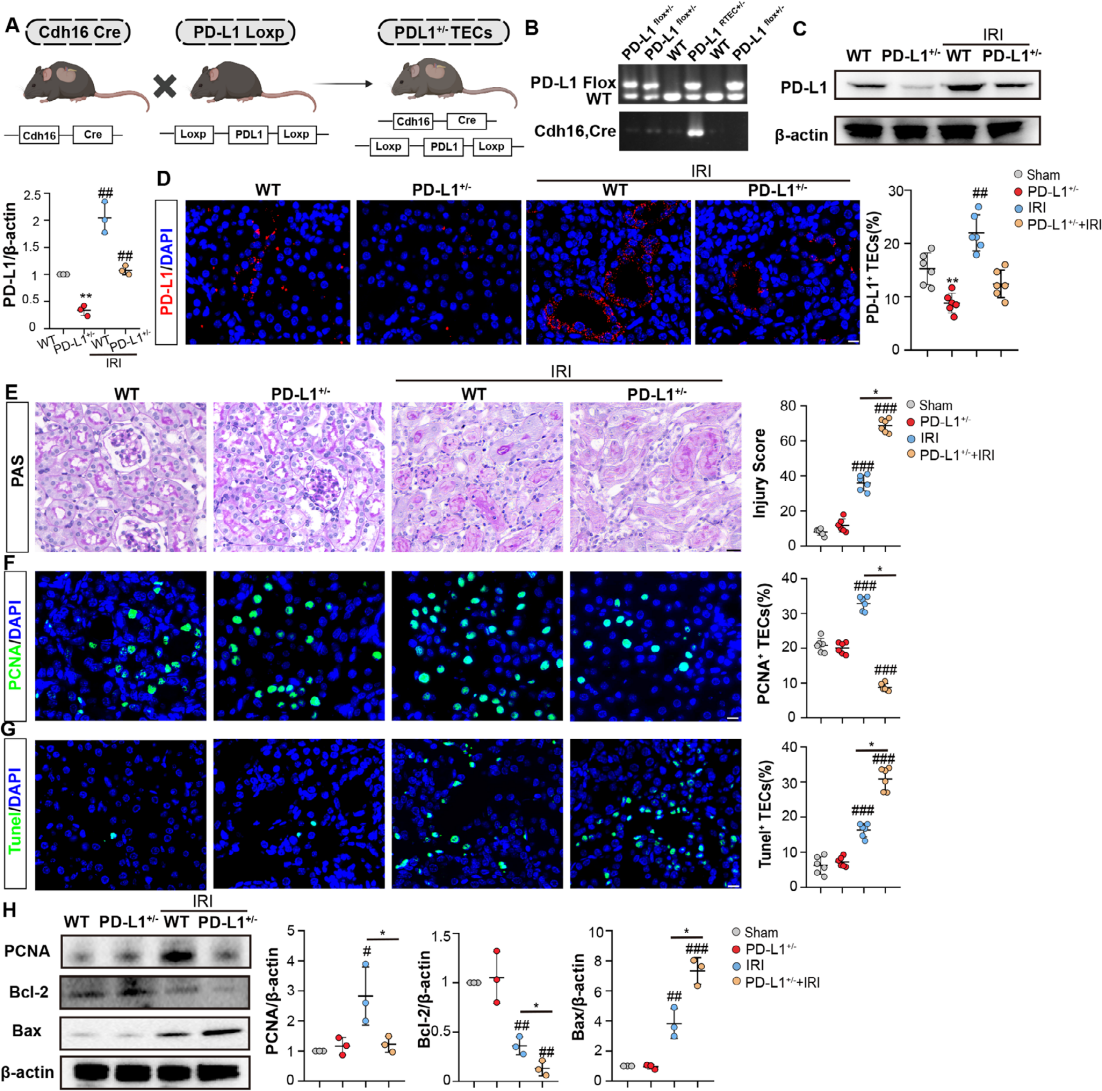
Selective knockdown of PD‐L1 in renal tubules exacerbates IRI‐induced AKI. (A) The flow chart of the construction of *PD‐L1^RTEC+/−^* mice. (B) Genomic DNA levels of PD‐L1 expression in WT, *PD‐L1^RTEC+/−^* mice and the WT and *PD‐L1^RTEC+/−^* mice model of renal IRI. (C) Western blot analysis of PD‐L1 protein levels in the above four groups. (D)Representative immunofluorescence images of the positive area of PD‐L1 staining in the above four groups. (E) Representative histopathology images of PAS staining in the above four groups. (F, G) Representative immunofluorescence images of PCNA and Tunel staining in the above four groups. (H) Western blot analysis of PCNA, Bcl‐2 and Bax protein levels in the above four groups. Scale bars, 20 µm. Data are presented as means ± SD. *P<0.05, **P<0.01 compared to sham group or IRI group; ^#^ *P* < 0.05, ^##^ *P* < 0.01, ^###^ *P* < 0.001 compared to *PD‐L1^RTEC+/−^* mice.

To demonstrate the protective role of PD‐L1 in vivo, tubule‐specific PD‐L1 knockdown (*PD‐L1^RTEC+/−^*) and knockout mice (*PD‐L1^RTEC‐/–^*) were generated (Figure 3A). Selective knockout of PD‐L1 in renal tubules exacerbates IRI‐induced AKI through histologic analysis in Figure S3A. EDU and Tunel staining further demonstrated reduced tubular cell proliferation and increased apoptosis in the *PD‐L1^RTEC‐/–^* group compared to the *PD‐L1^RTEC+/−^* group (Figure S3B–D). Notably, the anti‐PD‐L1 antibodies are well‐documented in anti‐cancer application and the PD‐L1 expression was mainly suppressed. To imitate the clinical effects, we utilized *PD‐L1^RTEC+/−^* mice in our following experiment. PD‐L1 deficiency was confirmed at the genomic DNA levels (Figure 3B), and Western blot showed an approximate 60% reduction in renal PD‐L1 expression (Figure 3C). Immunofluorescence analysis corroborated these results (Figure 3D). Interestingly, histologic analysis showed that PD‐L1 deficiency exacerbated IRI‐induced AKI, as evidenced by more severe tubular destruction and cast formation compared to controls (Figure 3E). PCNA and Tunel staining further demonstrated reduced tubular cell proliferation and increased apoptosis in the *PD‐L1^RTEC+/−^* group (Figure 3F,G). Consistently, Western blot analysis showed decreased expression of PCNA and Bcl‐2, along with elevated Bax levels in PD‐L1‐deficient kidneys (Figure 3H). Moreover, *PD‐L1^RTEC+/−^* mice exhibited significantly higher levels of BUN and Scr following cisplatin intervention compared to controls (Figure S4). The levels of Kim‐1 and Ngal expression was found elevated in the *PD‐L1^RTEC+/−^* group, while the infiltration of interstitial T cells showed no obvious difference in the *PD‐L1^RTEC+/−^* group, which further confirmed that PD‐L1 deficiency in TECs aggravates AKI likely through affecting tubular cells (Figure S5A–C). Therefore, these findings indicate that PD‐L1 deficiency in TECs aggravates AKI likely by impairing tubular cell repair.

### 2.4 PD‐L1 Alleviates DNA Damage by Interacting With Breast‐Cancer Susceptibility Gene 1 (BRCA1)

**FIGURE 4:**
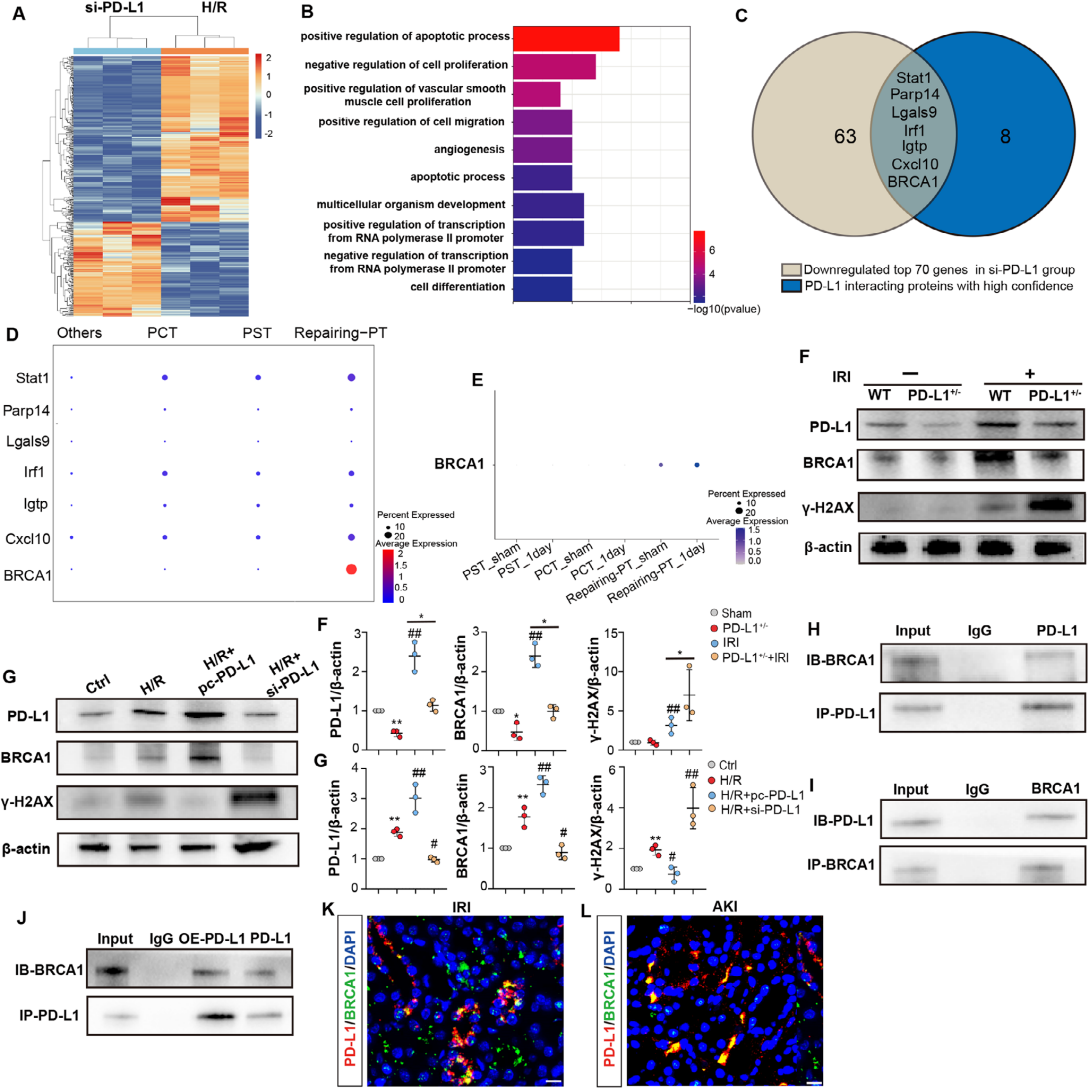
PD‐L1 alleviates DNA damage by interacting with breast‐cancer susceptibility gene 1. (A) Heatmap of RNA sequencing result on mTECs transfected with si‐PD‐L1 or a negative control after H/R treatment. (B) GO analysis showed enrichment pathways on mTECs transfected with si‐PD‐L1 after H/R treatment. (C) Protein‐protein interaction analysis predicted high‐confidence PD‐L1 interactors, and intersecting these genes with the top downregulated genes in the si‐PD‐L1 group. (D) The expression of the above intersecting genes in tubular subpopulations of our scRNA‐seq data from a uIRI‐induced AKI model. (E) The expression of Brca1 in sham mice or uIRI‐induced AKI model. (F) Western blot analysis of PD‐L1, BRCA1 and γ‐H2AX protein levels in the WT, *PD‐L1^RTEC+/−^* mice and the WT and *PD‐L1^RTEC+/−^* mice model of renal IRI. (G) Western blot analysis of PD‐L1, BRCA1 and γ‐H2AX protein levels in the transfecting mTECs with either si‐PD‐L1 or pc‐PD‐L1 after H/R treatment. (H, I) Immunoprecipitation analysis of PD‐L1 interaction with BRCA1 in mTECs lysates. (J) Immunoprecipitation analysis of PD‐L1 interaction with BRCA1 in the transfecting mTECs lysates with pc‐PD‐L1. (K,L) Representative immunofluorescence images of PD‐L1 and BRCA1 in mice model of renal IRI and AKI patients. Scale bars, 20 µm. Data are presented as means ± SD. *P < 0.05, **P < 0.01 compared to sham group, control or IRI group; ^#^ *P* < 0.05, ^##^ *P* < 0.01, ^###^ *P* <0.001 compared to *PD‐L1^RTEC+/−^* mice or mTECs after H/R treatment.

To investigate how PD‐L1 promotes tubular repair, we first performed RNA sequencing on mTECs transfected with si‐PD‐L1 or a negative control after H/R treatment to identify potential downstream targets. Differential gene expression was visualized in a heatmap (Figure 4A), and Gene Ontology (GO) analysis showed enrichment in pathways related to positive regulation of apoptotic process and negative regulation of cell proliferation following PD‐L1 knockdown (Figure 4B), consistent with our previous findings on PD‐L1's role in cell proliferation and apoptosis. Protein‐protein interaction analysis predicted 15 high‐confidence PD‐L1 interactors. By intersecting these with the top downregulated genes in the si‐PD‐L1 group, we identified 7 candidate genes: BRCA1, Stat1, Lgals9, Lgtp, Cxcl10, Parp14 and Irf1 (Figure 4C). Given PD‐L1's enrichment in TECs, we further examined the expression of these genes across tubular subpopulations using our scRNA‐seq data from a uIRI‐induced AKI model [20]. Notably, Brca1 was obviously expressed in the repairing‐proximal tubule (RT‐PT) population and upregulated at day 1 post‐uIRI (Figure 4D,E). This was further confirmed using the Kidney Interactive Transcriptomics database, which also showed Brca1 expression in the RT‐PT cluster at day 2 post‐uIRI (Figure S6). These results suggest that Brca1 may be a key downstream effector of PD‐L1 in promoting tubular repair during early AKI.

BRCA1 plays a crucial role in repairing DNA double‐strand breaks by favoring homologous recombination [21, 22]. Its deficiency leads to increased DNA damage [23]. Interestingly, Western blot analysis showed that PD‐L1 deficiency significantly reduced BRCA1 levels and increased γ‐H2AX expression, a well‐established marker of DNA double‐strand breaks, in kidneys following IRI (Figure 4F). Similar changes were observed in HK‐2 cells treated with PD‐L1 siRNA, whereas PD‐L1 overexpression markedly reversed these effects (Figure 4G). To elucidate whether PD‐L1 directly interacts with BRCA1, we performed immunoprecipitation assays. As shown in Figure 4H, BRCA1 was detected in mTECs lysates immunoprecipitated with an anti‐PD‐L1 antibody but not with control IgG. Conversely, immunoprecipitation with an anti‐BRCA1 antibody pulled down PD‐L1 (Figure 4I). Additionally, after PD‐L1 was over‐expressed in mTECs, BRCA1 was more detected in mTECs lysates immunoprecipitated with an anti‐PD‐L1 antibody (Figure 4J). Immunofluorescence staining confirmed the co‐localization of PD‐L1 and BRCA1 in injured kidneys of mice and AKI patient (Figure 4K,L). Moreover, molecular docking analysis revealed multiple potential interaction sites between PD‐L1 and BRCA1 in mice and human species (Figure S7A,B). Collectively, these findings indicated that PD‐L1 could protect injured TECs from DNA damage through interacting with BRCA1 and increasing BRCA1 expression.

### 2.5 CGA Peptide‐Engineered Extracellular Vesicles for Targeted PD‐L1 Delivery to Ischemic Kidneys

Our study demonstrated a protective role of PD‐L1 in TECs, highlighting its potential as a novel therapeutic target for AKI. However, effective strategies to restore PD‐L1 expression remain limited. Given the unique advantages of extracellular vesicles (EV) as drug carriers, we explored an EV‐based system for PD‐L1 delivery [24]. To achieve kidney‐specific targeting, we first evaluated two peptides, CLPVASC (CLP) and CGAREMC (CGA), previously reported to accumulate in healthy mouse kidneys. However, their distribution in injured kidneys remains unclear. We conducted *ex vivo* imaging of kidneys from uIRI mice intravenously injected with scramble, CLP, or CGA peptide. Notably, CGA peptide exhibited superior accumulation in injured kidneys (Figure S8A), with immunofluorescence confirming its localization primarily in renal tubules (Figure S8B). Therefore, CGA peptide was selected as the candidate peptide for targeted PD‐L1 delivery in the following study.

**FIGURE 5:**
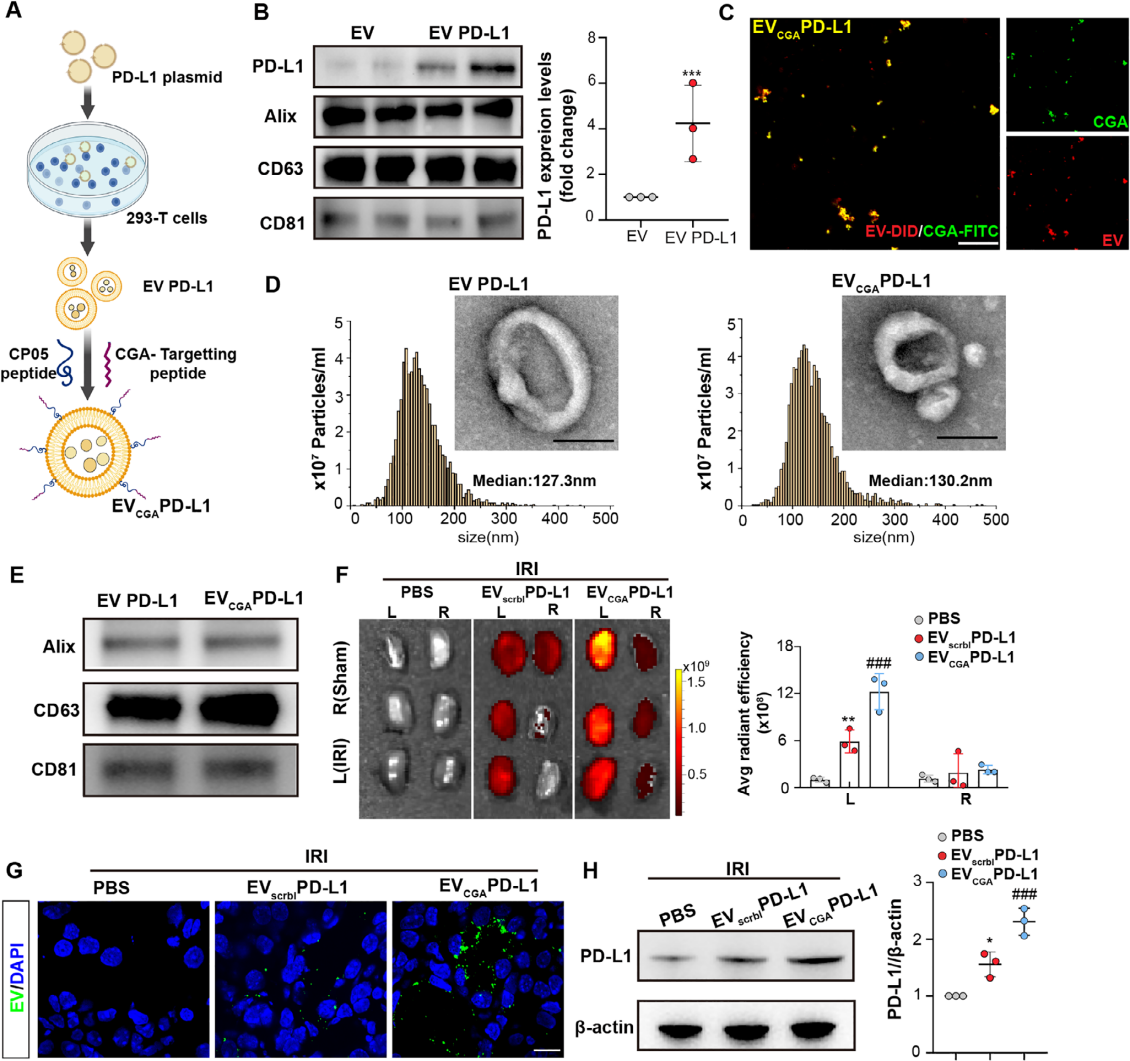
CGA peptide‐engineered extracellular vesicles for targeted PD‐L1 delivery to ischemic kidneys. (A) The flow chart of the construction of EV_CGA_PD‐L1. (B) Western blot analysis of PD‐L1, Alix, CD63 and CD81 protein levels in the EV and PD‐L1‐enriching EV (EV PD‐L1). (C) Representative immunofluorescence images of the colocalization of DID‐labeled EV and FITC‐labeled CGA peptide. (D) Nanoparticle tracking analysis and transmission electron microscope revealed the diameter and morphology of EV PD‐L1 and EV_CGA_PD‐L1. (E) Western blot analysis of Alix, CD63 and CD81 protein levels in EV PD‐L1 and EV_CGA_PD‐L1 group. (F) *Ex vivo* imaging of the kidneys in mice model of renal IRI treated with PBS, EV_scrbl_PD‐L1 and EV_CGA_PD‐L1. (G) Representative immunofluorescence images of EV in the above three groups. (H) Western blot analysis of PD‐L1 protein levels in the above three groups. Scale bars, 20 µm. Data are presented as means ± SD. *P < 0.05, **P < 0.01, *** *P* <0.001compared to sham group; ^###^ *P* < 0.001 compared to EV_scrbl_PD‐L1 group.

To develop the CGA peptide‐functionalized EV‐based PD‐L1 delivery system (EV_CGA_PD‐L1), we first transfected HEK293T cells with a PD‐L1 expression plasmid and isolated the resulting PD‐L1‐enriching EV. The CGA peptides were then conjugated onto these EV via CP‐05 peptides, a CD63‐targeting linker, to generate the EV_CGA_PD‐L1 system (Figure 5A). Western blot confirmed successful EV isolation and PD‐L1 loading, as indicated by the presence of PD‐L1, CD81, Alix, and CD63 (Figure 5B). Immunofluorescence demonstrated strong colocalization of DID‐labeled EV and FITC‐labeled CGA peptide, suggesting efficient CGA conjugation (Figure 5C). Nanoparticle tracking analysis (NTA) showed a slight increase in median EV diameter from 127.3 to 130.2 nm after CGA attachment, while transmission electron microscope (TEM) verified retention of the typical cup‐shaped morphology (Figure 5D). These data elucidated the successful construction of EV_CGA_PD‐L1. Moreover, the CD81, Alix, and CD63 protein levels also exhibited no apparent difference between EV_CGA_PD‐L1 and EV PD‐L1 (Figure 5E).

Next, we examined the kidney‐targeting ability of EV_CGA_PD‐L1 in a unilateral IRI model. *Ex vivo* imaging showed approximately a two‐fold increase in accumulation within the injured kidneys compared to the control EV_scrbl_PD‐L1(Figure 5F). Immunofluorescence further confirmed its preferential localization to injured kidneys (Figure 5G). Notably, Western blot showed significantly elevated PD‐L1 levels in EV_CGA_PD‐L1‐treated kidneys (Figure 5H), suggesting efficient PD‐L1 delivery. Histological examination of major organs showed no signs of toxicity (Figure S9). Together, these data demonstrate that CGA peptide markedly enhances EV_CGA_PD‐L1 homing to injured tubules, highlighting its potential as a targeted delivery vehicle for AKI treatment.

### 2.6 EV_CGA_PD‐L1 Protects Against Tubular DNA Damage During AKI

**FIGURE 6:**
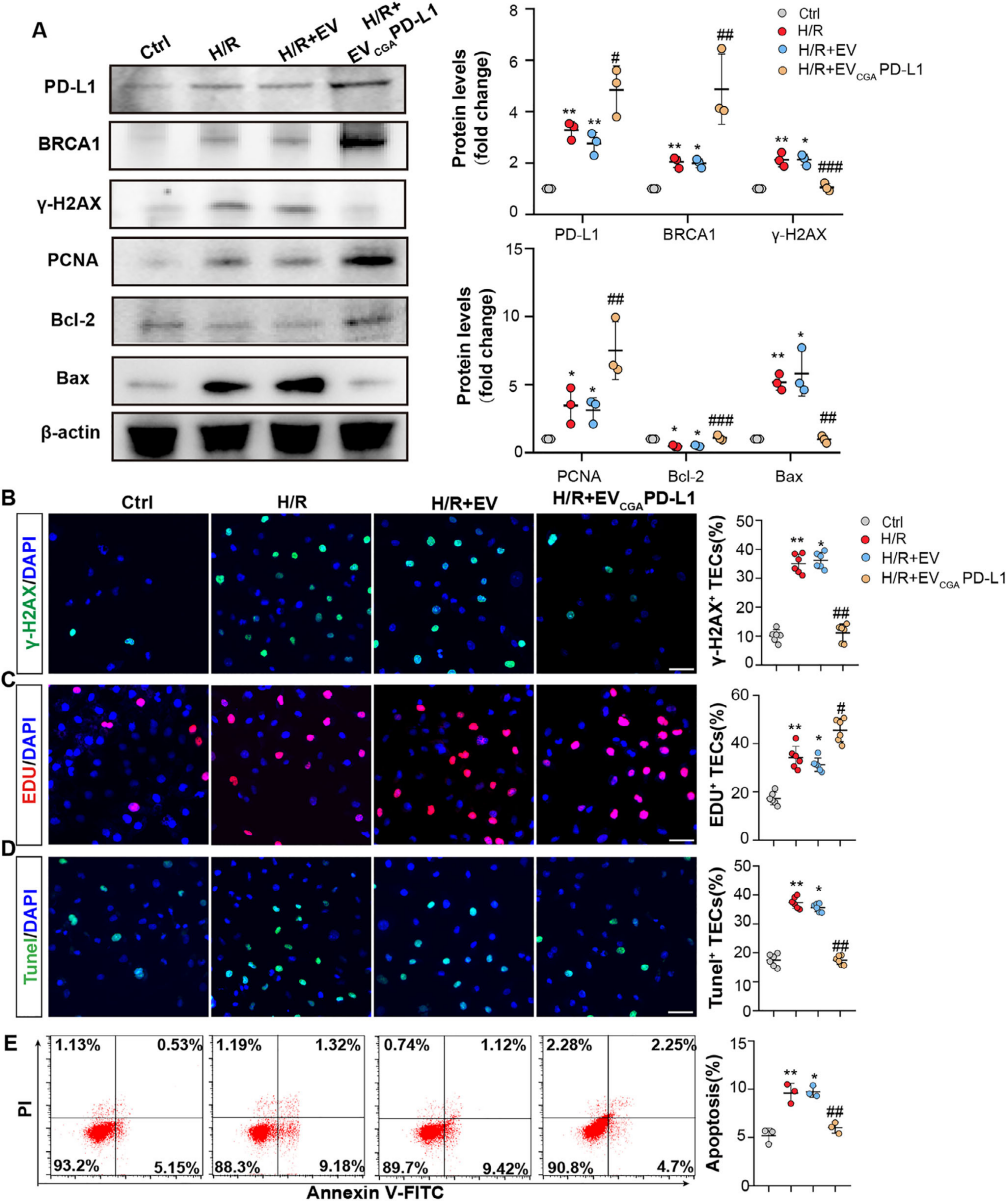
EV_CGA_PD‐L1 protects against tubular DNA damage after hypoxia/reoxygenation in vitro. (A) Western blot analysis of PD‐L1, BRCA1, γ‐H2AX, PCNA, Bcl‐2 and Bax protein levels in the mTECs with EV or EV_CGA_PD‐L1 intervention after H/R treatment. (B) Representative immunofluorescence images of γ‐H2AX in the mTECs with EV or EV_CGA_PD‐L1 intervention after H/R treatment. (C, D) EDU, Tunel staining analysis of cell proliferation and apoptosis in the mTECs with EV or EV_CGA_PD‐L1 intervention after H/R treatment. (E) Flow cytometer analysis of cell apoptosis in the mTECs with EV or EV_CGA_PD‐L1 intervention after H/R treatment. Scale bars, 20 µm. Data are presented as means ± SD. *P < 0.05, **P < 0.01 compared to sham group; ^##^ *P* < 0.01, ^###^ *P* < 0.001 compared to mTECs after H/R treatment.

We first evaluated the therapeutic efficacy of EV_CGA_PD‐L1 in vitro. Following H/R injury, EV_CGA_PD‐L1 remarkably enhanced PD‐L1 and BRCA1 expression while suppressing γ‐H2AX, indicating attenuated DNA damage (Figure 6A,B). As a result, EV_CGA_PD‐L1 promoted cell proliferation and inhibited apoptosis, as evidenced by increased PCNA and Bcl‐2 levels, decreased Bax expression, and corroborating findings from EDU (Figure 6C), Tunel (Figure 6D), and Annexin‐V/PI analysis (Figure 6E).

**FIGURE 7:**
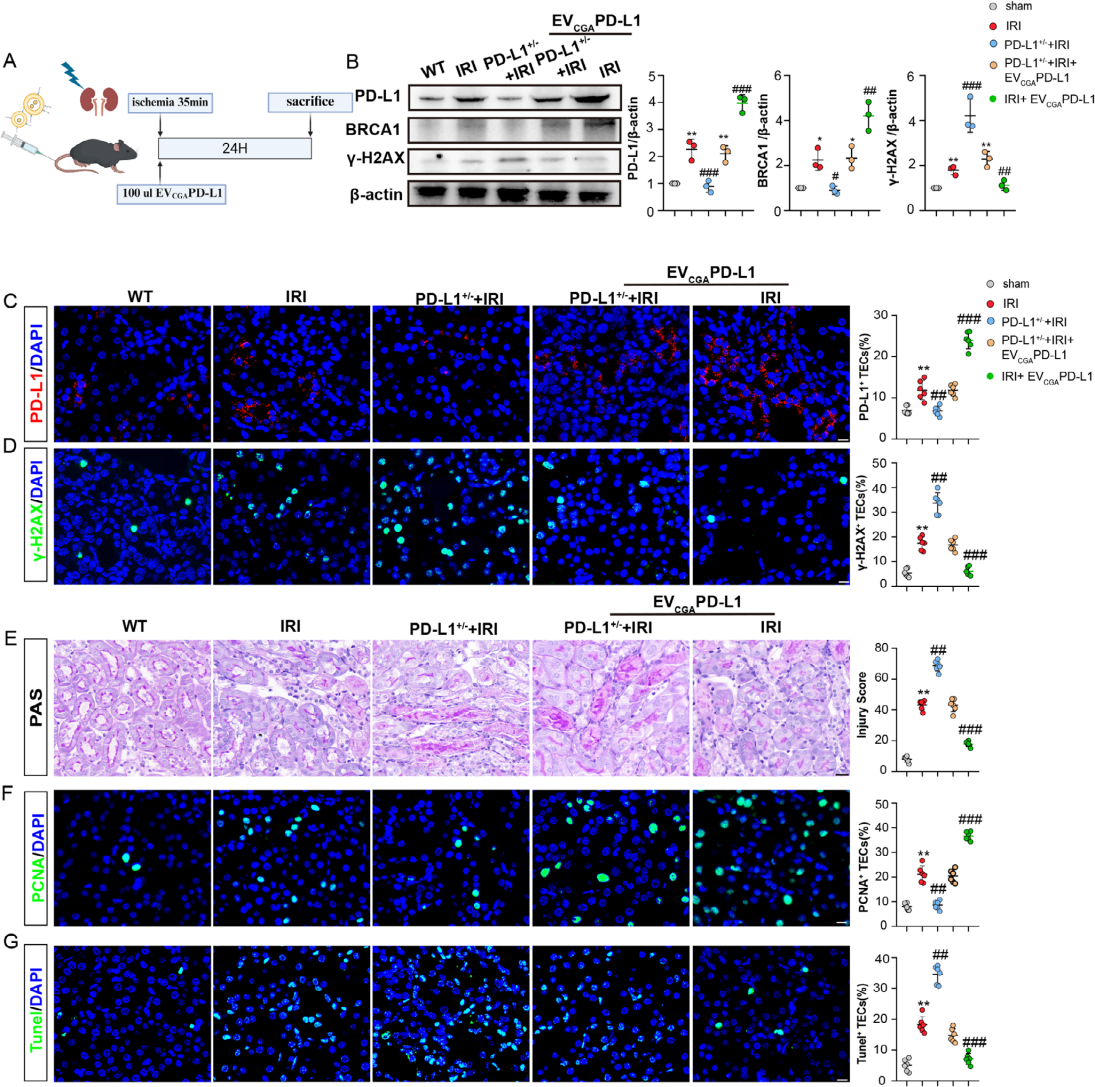
EV_CGA_PD‐L1 protects against tubular DNA damage during AKI in vivo. (A) The flow chart of the experimental. (B)Western blot analysis of PD‐L1, BRCA1 and γ‐H2AX protein levels in WT, the WT and *PD‐L1^RTEC+/−^* mice model of renal IRI, the WT and *PD‐L1^RTEC+/−^* mice model of renal IRI with EV_CGA_PD‐L1 intervention. (C) Representative immunofluorescence images of the positive area of PD‐L1 staining in the above five groups. (D) Representative immunofluorescence images of γ‐H2AX in the above five groups. (E) Representative histopathology images of PAS staining in the above five groups. (F, G) PCNA, Tunel staining analysis of cell proliferation and apoptosis in the above five groups. Scale bars, 20 µm. Data are presented as means ± SD. *P < 0.05, **P < 0.01 compared to sham group; ^##^ *P* < 0.01, ^###^ *P* < 0.001 compared to WT mice model of renal IRI.

Subsequently, we assessed the in vivo efficacy of EV_CGA_PD‐L1 in IRI‐induced AKI. EV_CGA_PD‐L1 was administered immediately after reperfusion for one time. Mice were sacrificed 24 h after EV_CGA_PD‐L1 injection (Figure 7A). In wild‐type mice, treatment with EV_CGA_PD‐L1 led to increased expression of PD‐L1 and BRCA1, along with reduced γ‐H2AX levels. Importantly, similar molecular benefits were observed in *PD‐L1^RTEC+/−^* mice, where EV_CGA_PD‐L1 restored PD‐L1 and BRCA1 expression and mitigated DNA damage (Figure 7B–D). PAS staining revealed that EV_CGA_PD‐L1 treatment preserved tubular structure and reduced TECs necrosis in both wild‐type and *PD‐L1^RTEC+/−^* mice (Figure 7E). Immunofluorescence staining further demonstrated improved TECs proliferation (Figure 7F) and apoptosis (Figure 7G) in treated groups. Taken together, our data suggest that PD‐L1 supplementation via EV_CGA_PD‐L1 can alleviate DNA damage of TECs, thereby facilitating tubular repair after AKI.

### 2.7 EV_CGA_PD‐L1 Mitigates AKI Through Direct TEC Protection Without Involving T Cells

**FIGURE 8:**
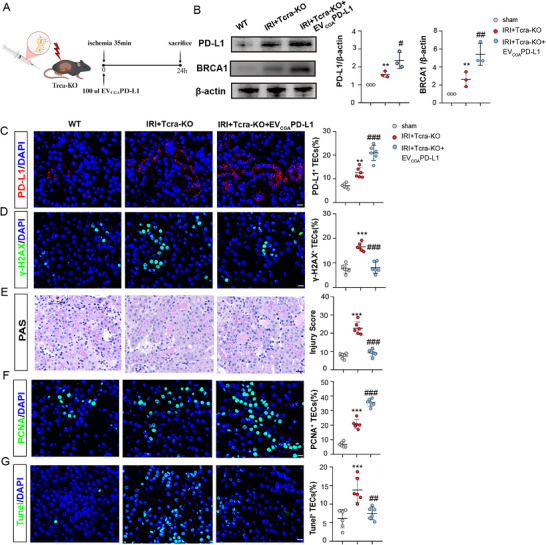
EV_CGA_PD‐L1 mitigates AKI through direct TEC protection without involving T cells. (A) The flow chart of the experimental. (B)Western blot analysis of PD‐L1 and BRCA1 protein levels in WT, the Tcra KO mice model of renal IRI with or without EV_CGA_PD‐L1 intervention. (C) Representative immunofluorescence images of the positive area of PD‐L1 staining in the above three groups. (D) Representative immunofluorescence images of γ‐H2AX in the above three groups. (E) Representative histopathology images of PAS staining in the above three groups. (F, G) PCNA, Tunel staining analysis of cell proliferation and apoptosis in the above three groups. Scale bars, 20 µm. Data are presented as means ± SD. **P < 0.01, *** *P* < 0.001compared to sham group; ^##^ *P* < 0.01, ^###^ *P* < 0.001 compared to the Tcra KO mice model of renal IRI.

To further demonstrate the T cell‐independent protective role of PD‐L1 on TECs, we employed a T‐cell receptor alpha constant region knockout (Tcra KO) mouse model of IRI‐induced AKI. These mice lack mature T cells, allowing us to evaluate the therapeutic efficacy of EV_CGA_PD‐L1 in the absence of T cell‐mediated immunity. As expected, EV_CGA_PD‐L1 significantly upregulated PD‐L1 and BRCA1 expression and mitigated DNA damage in Tcra KO mice subjected to IRI (Figure 8A–D). PAS staining revealed that EV_CGA_PD‐L1 effectively ameliorates IRI‐induced renal damage, including necrotic tubules, cast formation, and immune cell infiltration (Figure 8E). Additionally, EV_CGA_PD‐L1 enhanced TEC proliferation and reduced apoptosis (Figure 8F,G). Taken together, our data suggested that PD‐L1 alleviates renal injury after AKI through direct TEC protection without involving T cells (Figure S10).

## 3 Discussion

AKI is a common and serious clinical condition. Notably, cancer patients receiving anti‐PD‐L1 therapy are prone to AKI, prompting us to investigate the role and mechanism of PD‐L1 in AKI pathogenesis [25]. We demonstrated that PD‐L1 interacts with BRCA1 to protect TECs from DNA damage following ischemic AKI, independent of its canonical immunomodulatory role involving T cells. To harness this protective mechanism, we developed a CGA‐functionalized EV delivery system for targeted PD‐L1 delivery to injured TECs, offering a promising therapeutic strategy for AKI.

PD‐L1 maintains physiological homeostasis via immune‐dependent and independent mechanisms [26, 27]. As a well‐known immune checkpoint molecule, PD‐L1 regulates immune responses, especially in cancer immune evasion, immune tolerance, and immune suppression [12, 28]. Previous research has suggested that the injection of PD‐L1 fusion protein could ameliorate renal fibrosis in UUO mice model by increasing T cell exhaustion [29]. Hydroxychloroquine could alleviate renal fibrosis by evaluating PD‐L1 expression of TECs to enhance DCs immunosuppressive activity [30]. These studies suggest that PD‐L1 might serve as a protective role during the chronic stage after AKI. Emerging evidence indicates that PD‐L1 also exerts T cell‐independent functions through directly regulating cell death. For example, Xiao et al. revealed that PD‐L1 protects tumor‐associated dendritic cells from ferroptosis by downregulating SLC7A11 expression independent of T cells [31]. Additionally, Hou et al. identified a non‐immune checkpoint function of PD‐L1 and provide an unexpected concept that GSDMC/caspase‐8 mediates a non‐canonical pyroptosis pathway in cancer cells. [15]. These previous studies suggest that PD‐L1 can directly regulate cellular processes beyond immune modulation. However, its role in non‐immune cells during AKI remains unclear. In this study, we identified renal TECs as the primary site of PD‐L1 expression, which markedly increases during AKI. Using TEC‐specific PD‐L1 knockdown mice and in vitro knockdown and overexpression models, we demonstrated that PD‐L1 directly modulates TEC proliferation and apoptosis, thereby promoting tubular protection and repair.

PD‐L1 can modulate cellular phenotypes such as proliferation, metabolism, and stemness independent of T cells by regulating its downstream genes. A previous study revealed that PD‐L1 promotes non‐small cell lung cancer cell proliferation via the Gas6/MerTK signaling pathway [32]. Our study identified BRCA1 as a novel downstream target of PD‐L1 in TECs, showing that PD‐L1 can directly bind to BRCA1 and regulate its expression. BRCA1, a key DNA repair protein primarily expressed in the breast, prostate, and ovaries, facilitates genomic stability through DNA replication, double‐strand break detection, and signal transduction [33]. Currently, its role in kidney remains largely unexplored. Li et al. demonstrated that tubular BAP1 can stabilize BRCA1, thereby suppressing NF‐κB signaling and alleviating kidney injury in septic AKI [34]. Here, we found that BRCA1 is located in the cytoplasm of TECs and is upregulated during ischemic AKI, which could also be visualized in inhuman protein Atlas website. Moreover, PD‐L1 is found to interact with BRCA1 and increase BRCA1 expression, and plays a crucial role in reducing DNA damage of TECs, highlighting the PD‐L1‐BRCA1 axis as a promising therapeutic target for AKI.

DNA damage and the associated DNA damage response (DDR) are crucial drivers of AKI pathogenesis [35]. Following AKI, mediators such as reactive oxygen species trigger mitochondrial dysfunction and DNA double‐strand breaks, leading to DNA damage in TECs. Our previous studies demonstrated that in cisplatin‐induced AKI, microRNA‐155 promotes telomere dysfunction and DNA damage in TECs, while reduced CDK12 exacerbates TEC apoptosis and DNA damage [36, 37]. These findings highlight the pivotal role of TEC DNA damage in AKI. Notably, our current study reveals that DNA damage repair is a key mechanism by which PD‐L1 exerts renal protective effects. Specifically, PD‐L1 upregulates BRCA1 expression, thereby mitigating DNA damage in TECs during AKI. This is consistent with prior cancer research showing that PD‐L1 inhibition downregulates DDR‐related genes, enhancing tumor cell sensitivity to radiotherapy and chemotherapy [38]. Our findings provide new evidence that PD‐L1 promotes DNA repair in the kidney via an immune‐independent mechanism.

Based on our mechanistic findings, we propose that PD‐L1 supplementation may present a potential therapeutic strategy for AKI. While most studies focus on PD‐L1 inhibition for immune regulation, targeted delivery of PD‐L1 to injured kidneys remains underexplored. EVs have emerged as next‐generation drug delivery platforms due to their low toxicity and immunogenicity, and have been successfully employed to deliver small molecules, proteins, and nucleic acids for the treatment of various kidney diseases [39, 40]. However, a major challenge is that systemically administered EVs primarily accumulate in the mononuclear phagocyte system, limiting their renal targeting efficiency [41]. In our study, we identified kidney‐targeting peptides and found that the CGAREMC (CGA) peptide preferentially accumulates in IRI‐injured kidneys. We then developed a kidney‐targeted PD‐L1 delivery system by modifying EVs derived from gene‐edited HEK293T cells with CGA. This system effectively upregulated PD‐L1 and BRCA1 expression, reduced DNA damage in TECs and alleviated renal injury. Importantly, in both TEC‐specific PD‐L1 knockdown mice, and Tcra KO mice, EV‐mediated PD‐L1 supplementation also produced significant therapeutic benefits, further supporting its delivery specificity and efficiency, and a direct, T cell‐independent protective role of PD‐L1 in TECs.

In conclusion, this study uncovered a previously unrecognized role of PD‐L1 in facilitating tubular adaptive repair during AKI by directly interacting with BRCA1 to mitigate DNA damage— an effect independent of its canonical immunomodulatory function involving T cells. From a translational perspective, we developed a kidney‐targeted, EV‐based PD‐L1 supplementation strategy that demonstrated promising therapeutic efficacy in AKI. These findings not only reveal a novel facet of PD‐L1 in kidney disease but also position it as a promising therapeutic target for AKI.

## 4 Experimental Section

### 4.1 Animals

Renal tubular‐specific PD‐L1 knockdown (*PD‐L1^RTEC+/−^*) and knockout (*PD‐L1^RTEC‐/−^*) mice in a C57BL/6 background were generated by crossbreeding cdh16‐Cre with *PD‐L1* ^flox/flox^ mice. cdh16‐Cre mice were purchased from Cyagen Biosciences (Suzhou) Biotech Co., Ltd, while *PD‐L1* ^flox/flox^ mice were originally purchased from Shanghai Model Organisms Center, Inc. *PD‐L1^RTEC+/−^* mice at 8–9 weeks old with around 22–24g body weight were utilized in the following experiments. Age and weight‐matched C57BL/6 (wild‐type) mice were purchased from Vital River Laboratory Animal Technology Co., Ltd, and then were utilized in our further experiments. T‐cell receptor alpha constant region knockout (Tcra KO) mice were originally purchased from Cyagen Biosciences (Suzhou) Biotech Co., Ltd. All animal experiments were conducted in accordance with standard guidelines for the care and use of laboratory animals of the national institutes of health and approved by the Committee on the Ethics of Animal Experiments of Southeast University (No. 20220225043).

For the cisplatin‐induced AKI mouse model, a single intraperitoneal injection of cisplatin (18 mg/kg, Sigma) was administered, while control mice received an equivalent volume of normal saline. The unilateral ischemia‐reperfusion injury (uIRI)‐induced AKI model was established as previously described. Sham‐operated mice underwent the same surgical procedure without vascular clamping [42, 43]. All mice were sacrificed one day after treatment, and kidney samples were collected for further analyses.

### 4.2 Patient Samples

The collection of patient samples was approved by the Ethical Committee of Zhongda Hospital, Southeast University, and informed consent was obtained from all participants. A total of 13 AKI patients, diagnosed by serum creatinine, were enrolled in this study. Renal para‐carcinoma tissues from six individuals were set as normal controls. All samples were used for PD‐L1 immunostaining (Approval number:2017ZDSYLL107‐Y02).

### 4.3 Kidney Histology and Immunofluorescence

Briefly, mouse kidneys were fixed with 4% paraformaldehyde, embedded in paraffin, and sectioned into 4 µm thick slices for Periodic Acid‐Schiff (PAS) and immunofluorescence staining. Tubular injury was assessed semi‐quantitatively using a previously described histological scoring system [44]. Random kidney sections from mice were evaluated based on PAS staining results and the images were equally divided into 100 graticule grids. Histology of each graticule grid was semiquantitative evaluated: normal histology = 0, while immune cell infiltration, tubular cell swelling or necrosis, cast formation, brush border loss, tubular dilation or atrophy = 1.

Immunofluorescence staining was used to evaluate the position and expression of PD‐L1, PCNA, γ‐H2AX, BRCA1, AQP1, AQP2 and SLC12A3, which was performed on renal tissue section. The following antibodies were utilized for the immunofluorescence staining: PD‐L1 (ab205921, Abcam), γ‐H2AX (ab26350, Abcam), AQP1 (ab9566, Abcam), AQP2 (sc‐515770, Santa cruz), SLC12A3 (ab95302, Abcam), BRCA1 (20490R, Bioss), PCNA (GB11010, Service bio). Colorimetric Tunel Apoptosis Assay Kit (C1086, Beyotime) was used to assess the apoptosis percentage of TECs. Image Pro Plus image analysis system was then utilized to quantify the percentage of PD‐L1, PCNA, Tunel and γ‐H2AX positive percentage.

### 4.4 Cell Culture and Reagents

The kidneys were harvested from 4‐week‐old C57BL/6 mice, and primary mouse renal tubular epithelial cells (mTEC) were isolated as previously reported [42]. Briefly, the kidneys of mice were harvest, minced, and then digested in collagenase for 60mins at 37°C. The tubular tissues were then isolated, resuspended and seeded in the cell culture dishes. mTEC Cells were grown in cell culture dishes for about 5 days for the following experiment. Human renal tubular epithelial cells (HK‐2) and human embryonic kidney 293 T cells (HEK293‐T) cells were purchased from the Shanghai Institute of Biochemistry and Cell Biology (SIBCB), Chinese Academy of Sciences. Mouse and human renal tubular epithelial cells were cultured in DMEM/F12 medium containing 10% fetal bovine serum (FBS) and 100 mg/mL penicillin–streptomycin (P/S). HEK293‐T cells were cultured in RPMI‐1640 medium with 10% FBS and 100 mg/mL P/S. All cells were cultured at 37°C in a 5% CO_2_ incubator.

### 4.5 Cell Transfection

PD‐L1 siRNA, PD‐L1 plasmid, and negative control (NC) were designed and synthesized by Hanbio (Shanghai, China). The sequences of human PD‐L1 siRNA were: sense 5'‐ GGUGCCGACUACAAGCGAATT‐3'; antisense 5'‐UUCGCUUGUAGUCGGCACCTT‐3'. The sequences of mouse PD‐L1 siRNA were: sense 5'‐ GAGGUAAUCUGGACAAACATT‐3'; antisense 5'‐UGUUUGUCCAGAUUACCUCTT‐3'. HK‐2 or primary mTEC cells were transient transfected with PD‐L1 siRNA or PD‐L1 plasmid by using Lipofectamine 3000 (Invitrogen) according to the manufacturer's protocol. And the transfected cells were then incubated 48h after reoxygenation for the following procedure.

### 4.6 Flow Cytometry

Cell apoptosis was assessed using the Annexin V‐FITC apoptosis detection kit (KGA1102, KeyGEN). Briefly, cells were harvested, washed twice with PBS, and resuspended in binding buffer. The cells were then incubated with fluorescein isothiocyanate (FITC) – Annexin V and propidium iodide (PI) for 15 min at room temperature. Apoptosis was analyzed using a flow cytometer (FACScan; BD Biosciences), and the data were analyzed with FlowJo 10.8.

### 4.7 EDU and Tunel Staining Assay

EDU staining assay was performed according to the manufacturer's instructions (KGA9606, KeyGEN). Briefly, HK‐2 or mTEC cells were seeded into 24‐well plates. 10 µM EDU reagent was added into each well and then incubated at 37°C for 2 h. After incubation, cells were fixed with 4% formaldehyde for 15 mins and washed twice with PBS. Then the cells were incubated with 400 µL of 1×Click‐iT EDU reaction buffer for 30 mins, followed by two additional PBS washes. Nuclei were stained by Hoechst 33342. For the Tunel staining assay, it was performed with a Tunel staining kit (C1086, Beyotime) following the manufacturer's instructions. Briefly, HK‐2 or mTEC cells were seeded into 24‐well plates. Cells were washed twice with PBS and fixed with 4% formaldehyde for 30 mins. Then the cells were washed with PBS and incubated at room temperature for 5mins. After incubation, 50 µL Tunel reagent was added into the cells at 37°C for 1 h. The number of EDU or Tunel‐positive cells was counted in a blinded manner by utilizing a confocal microscope (FV3000, Olympus).

### 4.8 RNA Preparation and Quantitative Real‐Time Polymerase Chain Reaction (qRT‐PCR)

An RNA‐easy Isolation Reagent (R701, Vazyme) was used to extract total RNA from kidney tissues and TECs according to the manufacturer's instructions [19]. HiScript III RT All‐in‐one RT SuperMix Perfect for qPCR (R333, Vazyme) was utilized to synthesize complementary DNA. Real‐time PCR was conducted by using the ChamQ SYBR qPCR Master Mix (Q331, Vazyme). The primers were as follows: Human (Hu)PD‐L1 Forward primer: 5′‐TGGCATTTGCTGAACGCATTT‐3′; Reverse:5′‐TGCAGCCAGGTCTAATTGTTTT‐3′; Hu BRCA1 Forward primer: 5′‐GAAACCGTGCCAAAAGACTTC‐3′; Reverse: 5′‐CCAAGGTTAGAGAGTTGGACAC‐3′; Hu GAPDH Forward primer: 5′‐GGACCTGACCTGCCGTCTAG‐3′; Reverse: 5′‐GTAGCCCAGGATGCCCTTGA‐3′. Mouse (Mus) PD‐L1 Forward primer: 5′‐GTCACTTGCTACGGGCGTTT‐3′; reverse 5′‐CCACTAACGCAAGCAGGTCC‐3′; Mus BRCA1 Forward primer: 5′‐CGAGGAAATGGCAACTTGCCTAG‐3′; reverse 5′‐TCACTCTGCGAGCAGTCTTCAG ‐3′; Mus GAPDH Forward primer: 5′‐GGATGCTGCCCTTACCC‐3′; Reverse: 5′‐GTTCACACCGACCTTCACC‐3′. Relative gene expression was calculated by utilizing the 2^−ΔΔCT^ assay.

### 4.9 Western Blot

Total protein extraction and western blotting of tissues, cells, or extracellular vesicles were performed as previously described [45, 46]. The antibodies were performed as follows: anti‐PD‐L1 (ab205921, Abcam), anti‐BRCA1 (ab7817, Abcam), anti‐Bcl‐2 (sc‐7382, Santa Cruz), anti‐Bax (sc‐7480, Santa Cruz), anti‐PCNA (sc‐56, Santa Cruz), anti‐γ‐H2AX (ab26350, Abcam), anti‐Alix (sc‐53540, Santa Cruz), anti‐CD63 (sc‐5275, Santa Cruz), anti‐CD81 (ab109201, Abcam), anti‐β‐actin (Ab0035, Abways). The secondary antibodies were goat anti‐mouse HRP (FDM007, FDbio science) and goat anti‐Rabbit HRP (FDR007, FDbio science). Target proteins were normalized to β‐actin levels. Finally, an ECL chromogenic substrate was utilized to detect the fluorescent signals (BIO‐RAD, USA).

### 4.10 Preparation of PD‐L1 Overexpressing HEK293‐T‐Derived Extracellular Vesicles (EV PD‐L1)

To prepare EV PD‐L1, HEK293‐T cells were transfected with a PD‐L1 plasmid. Successful transfection was confirmed by qRT‐PCR and Western blot analysis. After transfection, the HEK293‐T cells were cultured in serum‐free medium for two days. The culture supernatants were collected for the extraction of EV PD‐L1. EV were isolated through gradient centrifugation and filtration. Briefly, the supernatants were centrifuged at 2000g for 20 mins, followed by 10000g for 30 mins at 4°C. The resulting supernatant was filtrated using a 0.22‐µm filter and ultracentrifuged at 100,000g for 90 mins (Beckman, USA). The purified EV PD‐L1 was resuspended in sterile PBS for the subsequent detection and application.

### 4.11 Conjugation of Targeted Peptide to EV PD‐L1

Chimeric peptides consisting of CGA (a kidney‐targeting peptide) and CP05 (a CD63‐binding peptide) were synthesized by GeneScript Co. Ltd with a purity exceeding 99%. A total of 20 µg of EV PD‐L1 was incubated with the CGA‐CP05 peptide overnight at 4°C, followed by washing in an ultracentrifuge tube with PBS and subsequent filtration using a 100‐kDa diafiltration tube (Millipore, USA) to eliminate unbound peptides. To verify successful conjugation of CGA‐CP05 to EV PD‐L1 (EV_CGA_PD‐L1), FITC‐labeled CGA‐CP05 was loaded onto DID‐labeled EV PD‐L1, and the resulting conjugates were visualized using a confocal microscope (FV3000, Olympus).

### 4.12 Molecular Docking

Protein Data Bank (https://www.rcsb.org/) was utilized to obtain protein structure for PD‐L1 and BRCA1. Briefly, the protein structure was managed through pymol2.3.0 software, including removing water molecules or counter ions. Then, hdock on‐line server (http://hdock.phys.hust.edu.cn/) was utilized to perform the protein–protein docking. Next, the docking complex was subjected to molecular dynamics. Molecular dynamics was performed using the sander module. After equilibration, the production molecular dynamics simulation was performed. All processing and analysis of the trajectory data were performed. The docking results were imported into Pymol 2.3.0 for analysis of the interaction patterns.

### 4.13 RNA‐seq Analysis

Primary mTECs were transfected with either PD‐L1 siRNA or scramble siRNA and subsequently subjected to hypoxia/reoxygenation (H/R) for 12 h [47]. Following treatment, total RNA was extracted from the cells and was checked for quantity by the Agilent 2100 Bioanalyzer System. cDNA libraries and next‐generation sequencing were performed at the Oebiotech Co. Ltd. Genes were considered significantly differentially expressed if the multiple tests corrected *P* value was <0.05.

### 4.14 Establishment of Hypoxia/Reoxygenation (H/R) Model and Treatment

mTEC or HK‐2 Cells were plated in a six‐well plate and cultured reached approximately 80% confluence for the H/R model. For the H/R group, cells were cultured in glucose‐free and FBS‐free culture medium and incubated under hypoxic conditions (1% O_2_, 94% N_2_, and 5% CO_2_) for 12 h. Additionally, cells were then transferred into the complete culture medium with oxygen for reoxygenation. In the treatment group, the cells were incubated with the CGA peptide‐functionalized EV‐based PD‐L1 (20 µg) for 12 h during hypoxia.

### 4.15 In Vivo Biodistribution and Injection of EV_CGA_PD‐L1

FITC‐labeled EV_CGA_PD‐L1 (100 µg) were injected intravenously into uIRI mice to observe the renal distribution of EV_CGA_PD‐L1 in vivo. Kidneys were excised 6 h post‐injection and then imaged by the IVIS spectrum imaging system (PerkinElmer). Additionally, EV_CGA_PD‐L1 was administered immediately after reperfusion for one time. Mice were sacrificed 24 h after EV_CGA_PD‐L1 injection.

### 4.16 Co‐Immunoprecipitation (Co‐IP)

To elucidate whether PD‐L1 directly interacts with BRCA1, Co‐IP assay was performed, First, mTECs lysates was lysed in radio immunoprecipitation assay (RIPA) lysis buffer. Protein concentrations were extracted and assessed by using the bicinchoninic acid (BCA) kit. Then the equivalent protein concentrations were incubated with protein G beads for 24h and washed with 0.5% Triton‐X‐100 for twice. The beads were collected by magnetic separation for the following step. The beads were incubated with anti‐PD‐L1 antibody or an anti‐BRCA1 antibody and control IgG for 24h. Then the washed with 0.5% Triton‐X‐100 for twice. Then the beads were collected by magnetic separation, washed by using 0.1 M glycine for 5 mins and then neutralized by adding 1 M Tris‐HCl. A total of protein was separated by sodium dodecyl sulfate polyacrylamide gel electrophoresis (SDS–PAGE) and then transferred to a polyvinylidene difluoride (PVDF) membrane. After blocking with ncmBlot blocking buffer overnight, the membrane was incubated with antibodies against PD‐L1 or BRCA1. Then the membrane was washed TBST and incubated with the secondary antibody. Finally, an ECL chromogenic substrate was applied to detect the fluorescent signals (BIO‐RAD).

### 4.17 Statistics Analysis

Statistical analyses were conducted using a t‐test or one‐way analysis of variance (ANOVA) in Prism 8.0 GraphPad Software. Data are presented as means ± SD. Statistical significance was defined as *P < 0.05.

## Author Contributions

B.W., B.‐C.L., W.J., and T.‐T.T. conceptualized the study. W.J. and T.‐T.T. prepared the manuscript. T.‐T.T., B.W., and B.‐C.L. edited the manuscript. W.J., T.‐T.T., W.‐J.N. and J.X.W. assembled figures. Y.L.Z., L.‐Y.‐Z.J., Q.Y., Z.‐L.L., Y.W., X.‐L.W., J.‐Y.S., M.‐Z.Z., X.‐J.O., X.A., J.X., W.J., and T.‐T.T. developed experimental strategy and analyzed results. B.W. and B.‐C.L. did project administration and supervision. W.J., T.‐T.T., B.W. and B.‐C.L. provided Funding acquisition.

## Funding

This work was supported by grants from the National Natural Science Foundation of China (82070735) and Clinical value of urinary exosome‑derived PLA2R protein levels in the diagnosis of membranous nephropathy (8590003946) to Bin Wang. This research was supported by additional grants from the National Natural Science Foundation of China (82230022, 82030024, 81720108007), National Key Research Programme of Ministry of Science and Technology (2022YFC2502503, 2018YFC130046) to Bi‐Cheng Liu. Natural Science Foundation of Jiangsu Province grant (BK20251135), Jiangsu Health Commission Foundation of China (LKM2025003), the Research Incubation Startup Fund of Jiangsu Province Geriatric Hospital (FHQD202505) to Wei Jiang. National Natural Science Foundation of China (82200772), Natural Science Foundation of Jiangsu Province grant (BK20220828) to Tao‐Tao Tang.

## Conflicts of Interest

The authors declare no conflicts of interest.

## Supporting information




**Supporting File**: advs75428‐sup‐0001‐SuppMat.pdf.

## Data Availability

The data that support the findings of this study are available from the corresponding author upon reasonable request.
