# Supplementary material for: T Cell‐Independent Role of PD‐L1 in Kidney Repair: Mitigation of Tubular DNA Damage via PD‐L1/BRCA1 Interaction Following AKI
Source: Adv Sci (Weinh). 2026 Apr 27;13(40):e75428. doi: 10.1002/advs.75428 (PMC13335584; doi:10.1002/advs.75428)
Supplement: Supplementary file 1 — Supporting File: advs75428‐sup‐0001‐SuppMat.pdf. [file ADVS-13-e75428-s001.pdf]

SUPPLEMENTARY MATERIALS:

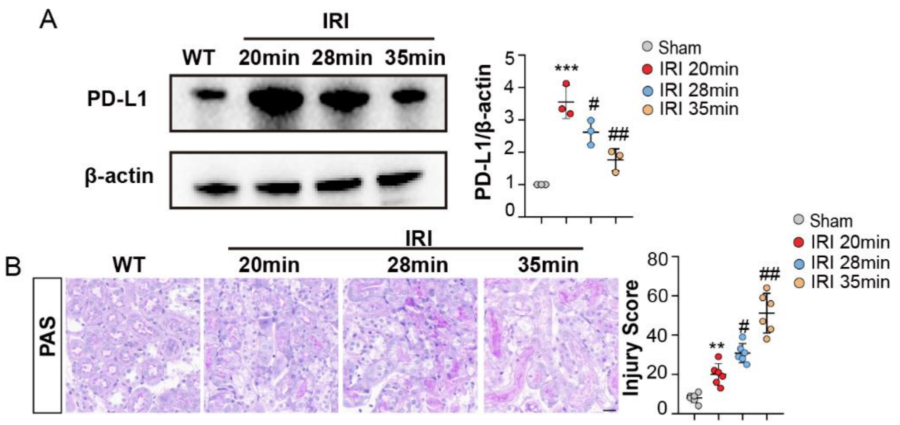

Supplementary Figure 1. **The prolonged ischemia led to more severe renal injury, accompanied by a gradual decline in PD-L1 expression.** (A)Western blot analysis of PD-L1 protein levels in the kidneys of sham and IRI models with increasing ischemia durations (20, 28, and 35 min). (B) Representative histopathology images of PAS staining in the above four groups. Data are presented as means ± SD. $^{**}P < 0.01$, $^{***}P < 0.001$compared to sham group; $^{\#}P < 0.05$, $^{\#\#}P < 0.01$ compared to IRI model with 20min.

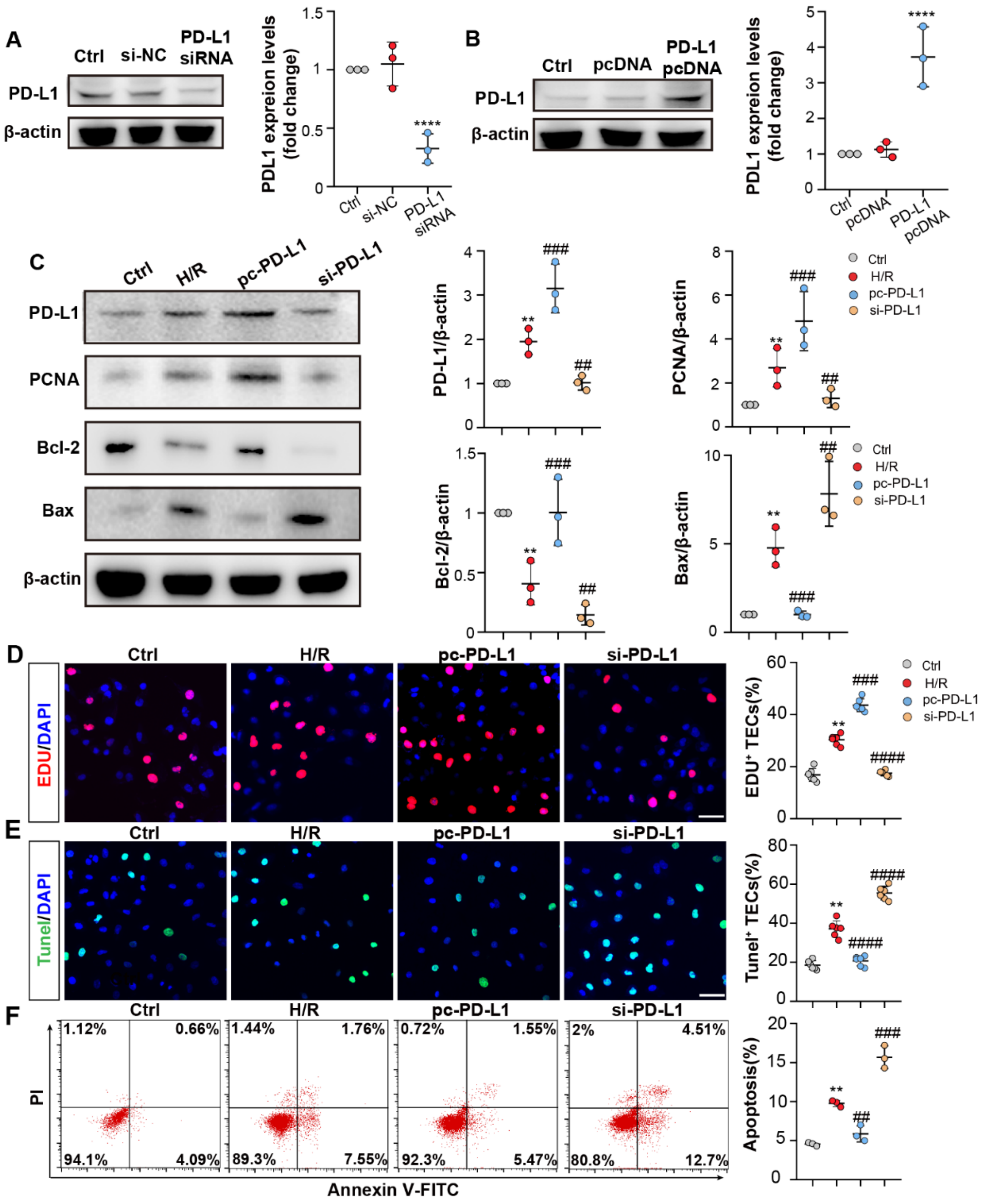

Supplementary Figure 2. **PD-L1 promotes the repair of injured HK-2 cells after hypoxia/reoxygenation *in vitro*.** (A, B) Western blot analysis of PD-L1 protein levels in the transfecting HK-2 cells with either si-PD-L1 or pc-PD-L1. (C)Western blot analysis of PD-L1, PCNA, Bcl-2 and Bax protein levels in the transfecting HK-2 cells with either si-PD-L1 or pc-PD-L1 after H/R treatment. (D, E) EDU, Tunel staining analysis of cell proliferation and apoptosis in the transfecting HK-2 cells with either si-PD-L1 or pc-PD-L1 after H/R treatment. (F) Flow cytometer analysis of cell apoptosis in the transfecting HK-2 cells with either si-PD-L1 or pc-PD-L1 after H/R treatment. Scale bars, 20μm. Data are presented as means ± SD. ***P*

< 0.01, **** P <0.0001 compared to control group; $^{\#\#}$ P <0.01, $^{\#\#\#}$ P <0.001, $^{\#\#\#\#}$ P <0.001 compared to HK-2 cells after H/R treatment.

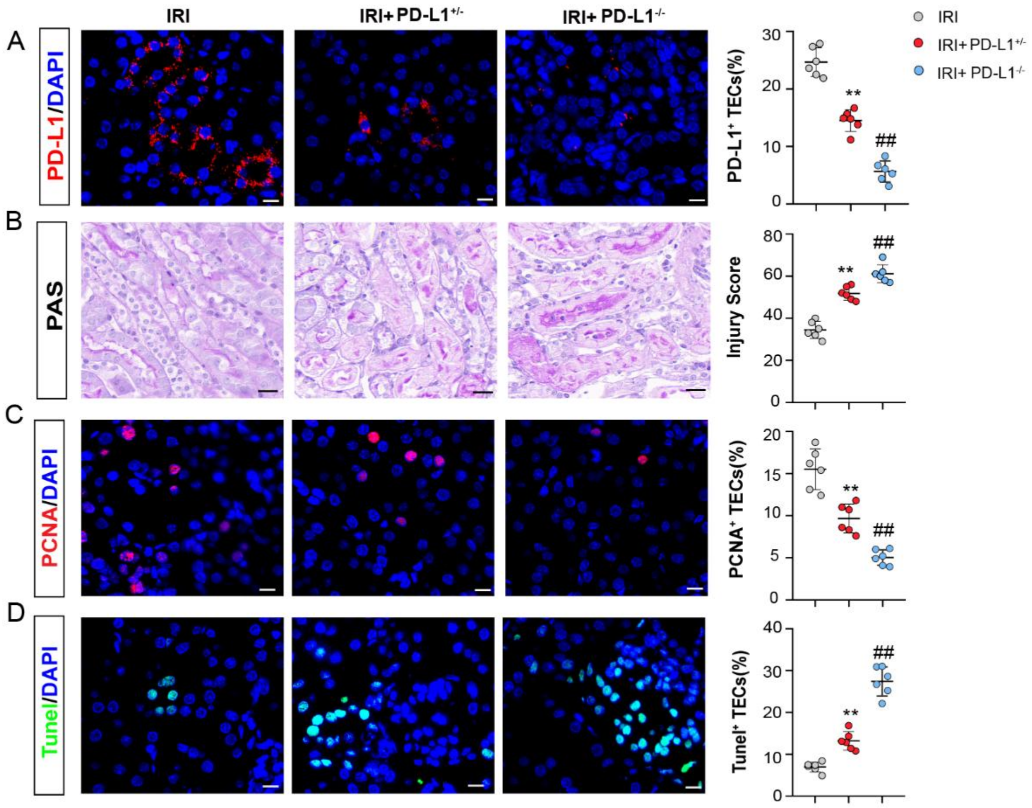

Supplementary Figure 3. **Selective knockout of PD-L1 in renal tubules exacerbates IRI-induced AKI.** (A) Representative immunofluorescence images of the positive area of PD-L1 staining in the WT, *PD-L1*$^{RTEC+/-}$and *PD-L1*$^{RTEC-/-}$murine model of renal IRI administration. (B) Representative histopathology images of PAS staining in the above three groups. (C, D) Representative immunofluorescence images of PCNA and Tunel staining in the above three groups. Scale bars, 20μm. Data are presented as means ± SD. **P < 0.01 compared to sham group; $^{\#\#}$ P <0.01 compared to *PD-L1*$^{RTEC+/-}$ mice.

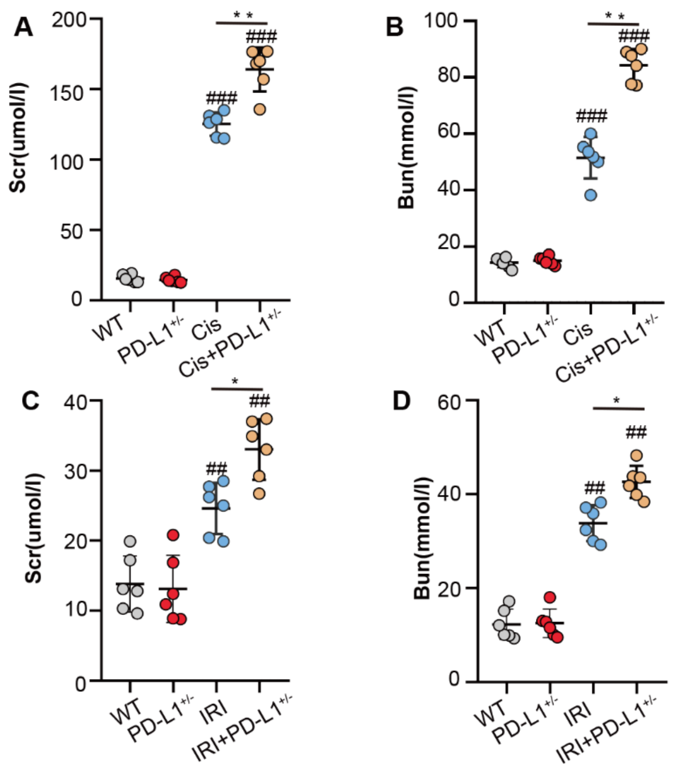

Supplementary Figure 4. ***PD-L1*$^{RTEC+/–}$ mice exhibited significantly higher levels of BUN and Scr following cisplatin or IRI intervention.** (A, B) The BUN and Scr levels in WT, *PD-L1*$^{RTEC+/–}$ mice and the WT and *PD-L1*$^{RTEC+/–}$ mice model following cisplatin intervention. (C, D) The BUN and Scr levels in WT, *PD-L1*$^{RTEC+/–}$ mice and the WT and *PD-L1*$^{RTEC+/–}$ mice model following IRI intervention. Data are presented as means ± SD. * P <0.05, **P < 0.01, compared to cis or IRI group; $^{\#\#}$ P <0.01,$^{\#\#\#}$$P$ <0.001 compared to *PD-L1*$^{RTEC+/–}$ mice.

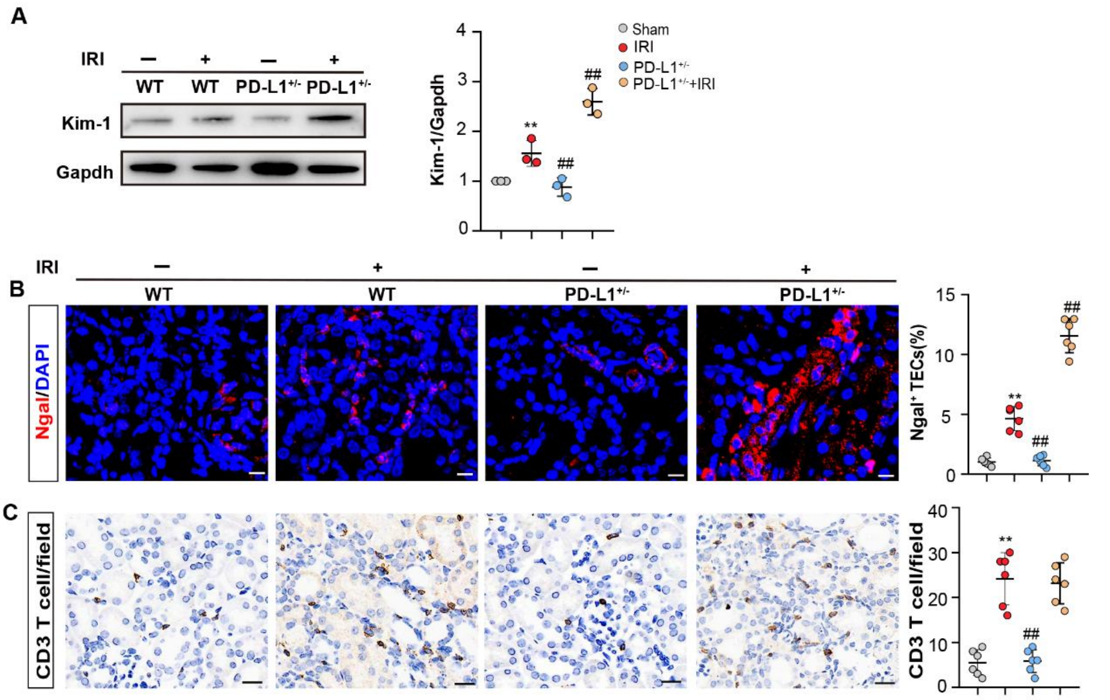

Supplementary Figure 5. **PD-L1 deficiency in renal tubules exacerbates IRI-induced renal injury.** (A) Western blot analysis of Kim-1 protein levels in WT, *PD-L1*$^{RTEC+/–}$ mice and the WT and *PD-L1*$^{RTEC+/–}$ mice model of renal IRI. (B)Representative immunofluorescence images of the positive area of Ngal staining in the above four groups. (C) Representative histopathology images of CD3 staining in the above four groups. Scale bars, 20μm. Data are presented as means ± SD. $^{**}P < 0.01$ compared to sham group; $^{\#}P$ <0.05, $^{\#\#}P$ <0.01, $^{\#\#\#}P$ <0.001 compared to *PD-L1*$^{RTEC+/–}$ mice.

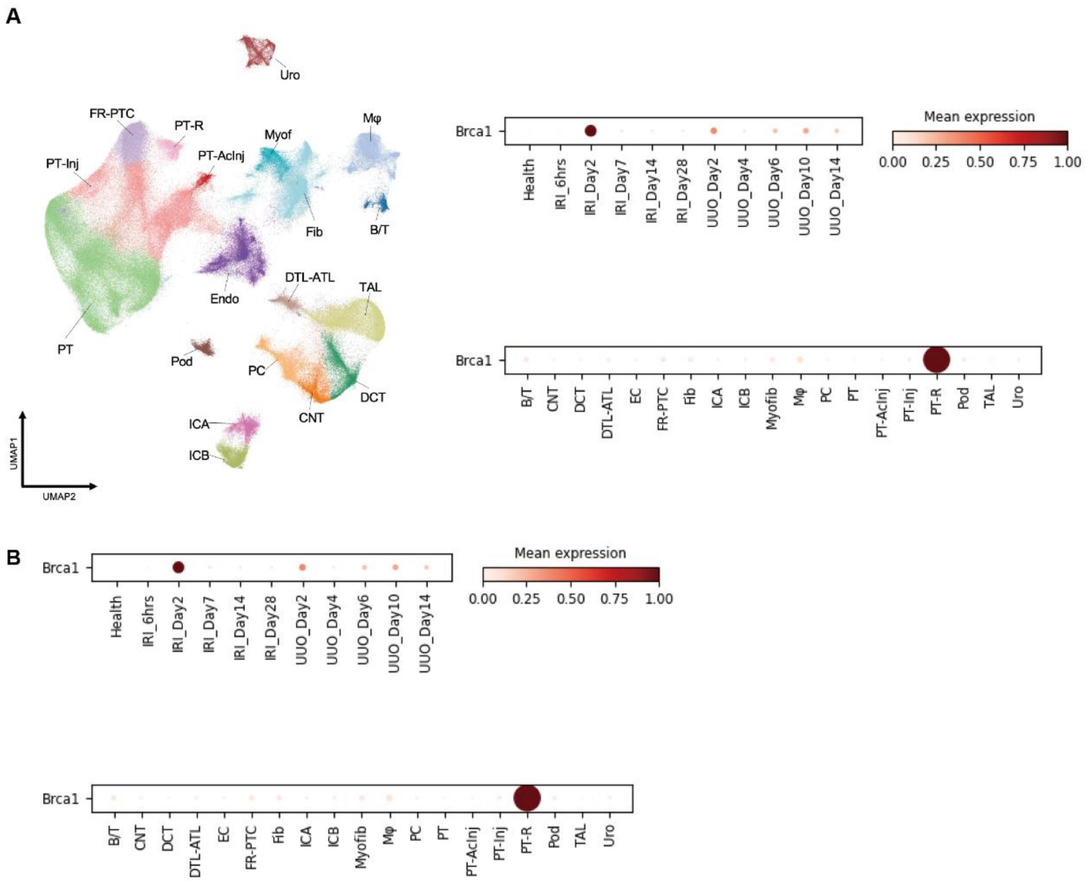

Supplementary Figure 6. **Brca1 was mainly expressed in the RT-PT cluster and increased at day 2 post-IRI in kidney interactive transcriptomics database.** (A, B) The expression of BRCA1 in each cell subtype of the kidney and IRI models with different ischemia durations by using the Kidney Interactive Transcriptomics database.

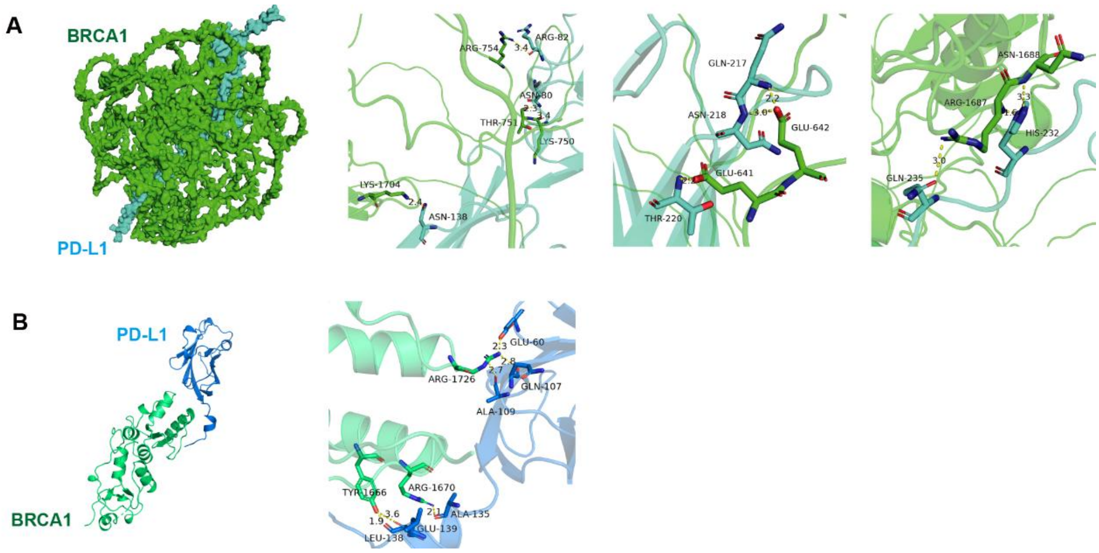

Supplementary Figure 7. Molecular docking analysis predicted the binding-sites between PD-L1 and BRCA1. (A) Molecular docking analysis of potential interaction sites between PD-L1 and BRCA1 （mice） (B) Molecular docking analysis of potential interaction sites between PD-L1 and BRCA1 （human） .

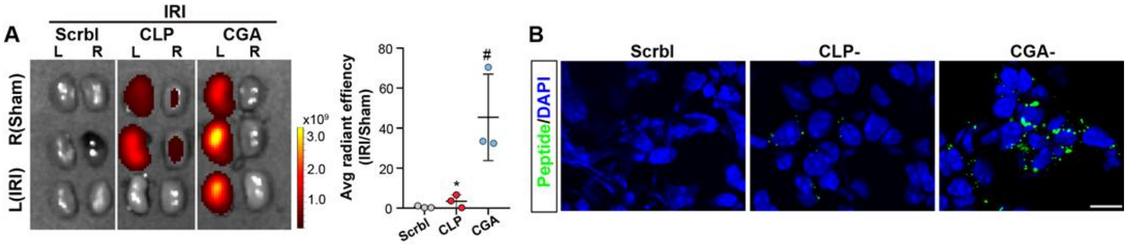

Supplementary Figure 8. **CGA peptide exhibited superior accumulation in injured kidneys.** (A) *Ex vivo* imaging of the kidneys from IRI mice intravenously injected with scramble, CLP, or CGA peptide. (B) Representative immunofluorescence images of peptide in the above three groups. Data are presented as means ± SD. *P < 0.05 compared to scramble group; [#] P <0.05 compared to EV_{CLP}PD-L1 group.

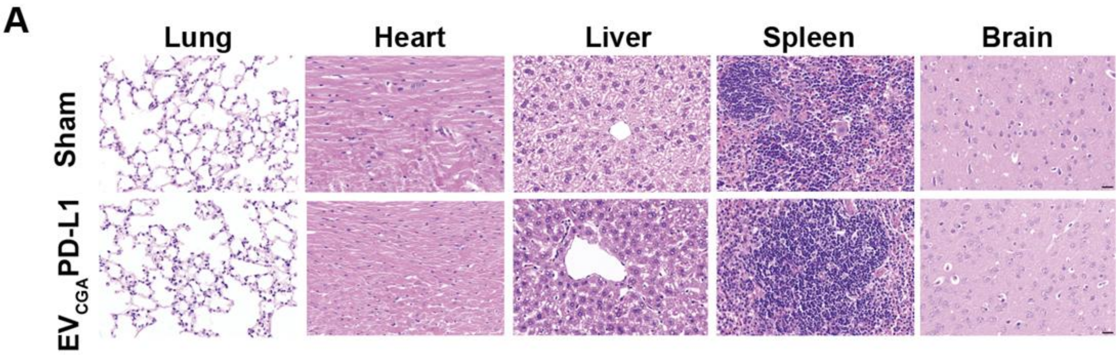

Supplementary Figure 9. **Histological examination of major organs showed no signs of toxicity in EVCGAPD-L1-treated kidneys.** (A) Histological examination of lung, heart, liver, spleen and brain organ showed no signs of toxicity in EVCGAPD-L1-treated group.

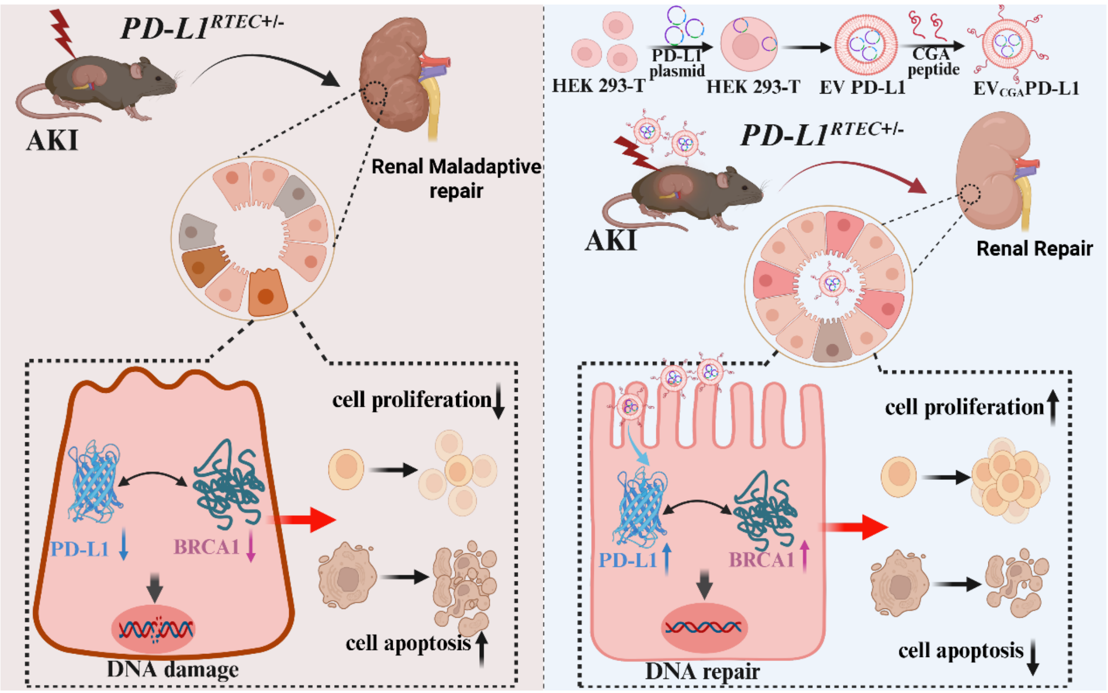

Supplementary Figure 10. **Working models propose a novel function of PD-L1 in promoting adaptive TEC repair through BRCA1 interaction following AKI, independent of its canonical immunomodulatory function of T cells.** We demonstrated that PD-L1 could promote tubular adaptive repair during AKI by directly interacting with BRCA1 to mitigate DNA damage, and a kidney-targeted, EV-based PD-L1 supplementation strategy that demonstrated promising therapeutic efficacy in AKI.
